# Combining association with linkage mapping to dissect the phenolamides metabolism of the maize kernel

**DOI:** 10.3389/fpls.2024.1376405

**Published:** 2024-04-12

**Authors:** Min Deng, Qingping Zeng, Songqin Liu, Min Jin, Hongbing Luo, Jingyun Luo

**Affiliations:** ^1^ College of Agronomy, Hunan Agricultural University, Changsha, China; ^2^ National Key Laboratory of Crop Genetic Improvement, Huazhong Agricultural University, Wuhan, China

**Keywords:** maize, phenolamides, association analysis, linkage mapping, protein-protein network

## Abstract

Phenolamides are important secondary metabolites in plant species. They play important roles in plant defense responses against pathogens and insect herbivores, protection against UV irradiation and floral induction and development. However, the accumulation and variation in phenolamides content in diverse maize lines and the genes responsible for their biosynthesis remain largely unknown. Here, we combined genetic mapping, protein regulatory network and bioinformatics analysis to further enhance the understanding of maize phenolamides biosynthesis. Sixteen phenolamides were identified in multiple populations, and they were all significantly correlated with one or several of 19 phenotypic traits. By linkage mapping, 58, 58, 39 and 67 QTLs, with an average of 3.9, 3.6, 3.6 and 4.2 QTLs for each trait were mapped in BBE1, BBE2, ZYE1 and ZYE2, explaining 9.47%, 10.78%, 9.51% and 11.40% phenotypic variation for each QTL on average, respectively. By GWAS, 39 and 36 significant loci were detected in two different environments, 3.3 and 2.8 loci for each trait, explaining 10.00% and 9.97% phenotypic variation for each locus on average, respectively. Totally, 58 unique candidate genes were identified, 31% of them encoding enzymes involved in amine and derivative metabolic processes. Gene Ontology term analysis of the 358 protein-protein interrelated genes revealed significant enrichment in terms relating to cellular nitrogen metabolism, amine metabolism. GRMZM2G066142, GRMZM2G066049, GRMZM2G165390 and GRMZM2G159587 were further validated involvement in phenolamides biosynthesis. Our results provide insights into the genetic basis of phenolamides biosynthesis in maize kernels, understanding phenolamides biosynthesis and its nutritional content and ability to withstand biotic and abiotic stress.

## 1 Introduction

Maize (*Zea mays* L.) is the world’s most widely grown crop for staple foods, animal feed, biofuel and other industrial raw materials. By 2050, it is estimated that the human population will reach 9 billion (FAO, 2009). Therefore, increasing maize yield while providing additional nutritional value is essential to meet the growing nutritional needs of a large global population (Casas et al., 2014; Jin et al., 2017).

Phenolamides (PAs) are important secondary metabolites in plant species. They are often referred to as hydroxycinnamic acid amides (HCAA) or phenylamides. PAs are primarily found in reproductive organs and seeds of plants and are believed to be either products of polyamine catabolism or stored forms of polyamines or phenols (Bassard et al., 2010). Chemically, PAs are conjugates of various hydroxycinnamic acids (such as coumaric, caffeic and ferulic acids) with mono/polyamines (such as tyramine, putrescine, spermine and spermidine) (Tanabe et al., 2016; Peng et al., 2016). PAs are highly diverse natural products identified in a broad number of plant species, such as barley (*Hordeum vulgare*) (Pihlava, 2014; Van Zadelhoff et al., 2022), rice (*Oryza sativa*) (Tanabe et al., 2016; Peng et al., 2016; Dong et al., 2015), maize (*Zea mays*) (Wen et al., 2014), tomato (*Solanum lycopersicum*) (Roumani et al., 2022), tobacco (*Nicotiana attenuata*) (Onkokesung et al., 2012; Ullmann-Zeunert et al., 2013; Figon et al., 2021), Arabidopsis (*Arabidopsis thaliana*) (Fellenberg et al., 2012), tea (*Camellia sinensis*) (Wang et al., 2023) and potato (*Solanum tuberosum*) (Yogendra et al., 2017). PAs are involved in various biological activities in plants (Wang et al., 2020). They play an important role in plant defense responses against pathogens and insect herbivores (Gaquerel et al., 2013, 2013, 2014; Figon et al., 2021; Fang et al., 2022; Xu et al., 2022). They are also suggested to play roles in sulfur starvation, heat shock, salt stress, protection against UV irradiation and floral induction and development (Guo et al., 2003; Demkura et al., 2010; Onkokesung et al., 2012).

Phenolamides biosynthesis is one of most intensively researched fields of plant secondary metabolism, and the synthesis steps of phenolamides are generally conserved across different plant species, indicating a high degree of evolutionary conservation (Bassard et al., 2010). The key step in phenolamide biosynthesis is catalyzed by N-hydroxycinnamoyltransferase (HT), which acts at the entry point of the pathway (Petersen, 2016; Roumani et al., 2021). Several HTs have been cloned in various plant species, including tyramine hydroxycinnamoyl transferases (THTs) in potato, tomato and rice (Schmidt et al., 1999; Shen et al., 2021), putrescine hydroxycinnamoyl transferases (PHTs) in rice and maize (Wen et al., 2014; Fang et al., 2021), serotonin N-hydroxycinnamoyl transferases (SHTs) in pepper (Kang et al., 2006), tryptamine hydroxycinnamoyl transferases (TBTs) in rice (Peng et al., 2016), and agmatine hydroxycinnamoyl transferases (AHTs) in Arabidopsis and rice (Muroi et al., 2009; Peng et al., 2016). The BAHD acyltransferase family, named after its members benzyl alcohol O-acetyltransferase (BEAT), anthocyanin O-hydroxycinnamoyl transferase (AHCT), anthranilate N-hydroxycinnamoyl/benzoyl transferase (HCBT), and deacetylvindoline 4-O-acetyltransferase (DAT), is also involved in phenolamide biosynthesis (St-Pierre and De Luca, 2000). Enzymes from branches I, IV, and V of the BAHD acyltransferase family have been identified in phenolamide biosynthesis (Peng et al., 2016). Agmatine coumaroyl transferases (ACTs) have been identified in *Brachypodium distachyon* (Carere et al., 2018), Arabidopsis (Muroi et al., 2009) and barley (Burhenne et al., 2003). Spermidine hydroxycinnamyl transferase, which produces hydroxycinnamoyl spermidines, has been found in Arabidopsis, tobacco leaves, eggplant and rice (Grienenberger et al., 2009; Luo et al., 2009; Onkokesung et al., 2012; Dong et al., 2015; Peng et al., 2016). Putrescine hydroxycinnamoyl transferases have been identified in both dicots (*Nicotiana*) and monocots such as maize and rice (Onkokesung et al., 2012; Wen et al., 2014; Chen et al., 2014b). Despite the identification of several genes involved in phenolamide biosynthesis, the process is still not well understood, particularly in maize. Further research is needed to unravel the complete biosynthetic pathway and regulatory mechanisms of phenolamides in maize and other plant species.

In recent years, the rapid development of metabolomics and the use of different populations for genetic mapping have provided us an unprecedented insight into the regulation of the abundance of multiple chemical components in plants (Wen et al., 2016). Based on the spatiotemporal distribution characteristics of PAs with the natural genetic diversity of plants, several new PA biosynthetases were successfully identified in rice and maize (Wen et al., 2014; Dong et al., 2015). Two spermidine N-hydroxycinnamoyl transferases, LOC_Os12g27220 and LOC_Os12g27254, responsible for the biosynthesis of spermidine-containing PAs in rice have been recently reported by GWAS (Dong et al., 2015). Several additional rice genes were associated with the condensation of putrescine and agmatime with hydroxycinnamoyl-CoA substrates in GWAS experiments (Chen et al., 2014b).

Previously, comprehensive metabolic profiling using liquid chromatography tandem mass spectrometry (LC–MS/MS) was carried out in mature maize kernels from association panel and RIL populations (Wen et al., 2014, 2015, 2016). Combined linkage analysis and GWAS were carried out on the resultant datasets, which led to the identification of a variety of loci involved in multiple biosynthetic pathways (Wen et al., 2014, 2016). Here, we combined genetic mapping, metabolite profiling from these previous studies and protein regulatory network analysis to further enhance the understanding of the maize phenolamides pathway. Correlation between phenolamides and agronomic performance (Yang et al., 2014), GWAS, linkage mapping, protein regulatory network, and bioinformatics analysis of candidate genes were conducted in the current study. These results provide new insights for understanding phenolamides biosynthesis and its nutritional content and ability to withstand biotic and abiotic stress.

## 2 Materials and methods

### 2.1 Genetic materials and field trials

The metabolic data used in this study were obtained from genetic materials, including an association mapping panel with 368 lines (referred to as AMP hereafter) for GWAS and two recombinant inbred line populations (RILs; BB, F_9_ RIL B73/By804, and ZY, F_10_ RIL Zong3/Yu87-1) for linkage analysis as described previously (Wen et al., 2014; Pan et al., 2016), BB and ZY RIL populations was derived from a single F_1_ plant and was developed through self-pollination and single seed descent for nine and ten generations, respectively. Maize kernels of AMP were planted in Yunnan (Kunming, E 102°30′, N 24°25′, referred to as AMPE1) and Chongqing (E 106°50′, N 29° 25′, referred to as AMPE2) in March 2011, the 197 BB RIL population was planted in Hainan (Sanya; E 109°519, N 18°259) in October 2010 (referred to as BBE1) and Henan (Zhengzhou; E 113°429, N 34°44′) in June 2011 (referred to as BBE2), and the 197 lines of the ZY RIL population were planted in Yunnan (Kunming; E 102°309, N 24°259; referred to as ZYE1) and Henan (Zhengzhou; E 113°429, N 34°449; referred to as ZYE2) in March and June 2011, respectively. An incomplete block design was used for the field trials of all the inbred lines, including AMP and two RIL populations, and a single replicate was conducted in each environment. All lines were self-pollinated, and five ears were harvested from each plot at maturity and air-dried and shelled. For each line, ears from five plants were harvested at the same maturity and bulked. Twelve well-grown kernels were randomly selected from the harvested ears and bulked for grinding (Wen et al., 2014; Deng et al., 2020).

### 2.2 Metabolic data, genotype and expression data

Samples from each line of AMP and RIL populations were extracted before analysis using an LC–ESI–MS/MS system, more details information were provided in previous study (Wen et al., 2014). The genotype data was used in present study obtained from the Maizego database (http://www.maizego.org/Resources.html) consisted of 1.25 million SNP (B73_RefGen_v2) that covered the whole maize genome, with a minimum allele frequency ≥ 0.05 (Liu et al., 2017). The two RIL populations were also genotyped by the Illumina MaizeSNP50 BeadChip, and high-density linkage maps were constructed with 2496 and 3071 unique bins for BB and ZY, respectively (Pan et al., 2016; Xiao et al., 2016). The expression data of 28 769 genes were obtained by RNA sequencing from kernel of five immature ears of 368 maize inbred lines were collected 15 days after self-pollination for RNA extraction (Fu et al., 2013; Li et al., 2013).

### 2.3 Genetic mapping

A genome-wide association study (GWAS) was conducted for maize kernel phenolamides. To test the statistical associations between genotype and phenotype, a mixed linear model was used to account for the population structure and relative kinship (Li et al., 2013). Considering the maker number in the present study is 1.25 million, many of them should be in linkage disequilibrium. The effective number of independent markers (N) was calculated using the GEC software tool (Li et al., 2012). Suggestive (1/N) P value thresholds were set to control the genome-wide type 1 error rate. The suggestive value was 2.04E-06 for the whole population and was used as the cutoff (Deng et al., 2017). The P value of each SNP was calculated using Tassel3.0. For all traits, the lead SNP (SNP with the lowest p value) at an associated locus and its corresponding candidate genes in or near (within 100 kb up- and downstream of the lead SNP) known genes were reported. If the associated SNPs were not in or near an annotated phenolamides metabolism gene, the closest of the lead SNP candidate gene was considered the most likely candidate gene (Deng et al., 2017). The physical locations of the SNPs were based on B73 RefGen_v2.

Linkage mapping was conducted using composite interval mapping (CIM) implemented in Windows QTL Cartographer V2.5 (Zeng and Kao, 1999; Wang et al., 2010) for all phenolamides measured in the maize kernels of the two RIL populations. The methods followed the Windows QTL Cartographer V2.5 user manual. Zmap (Model 6) with a 10 cM window and a walking speed of 0.5 cM was used. For each trait, a uniform threshold for significant QTLs was determined by 500 permutations (*p* = 0.05). The parameter was set as default. A 2.0 LOD-drop confidence interval was used for each QTL as described.

Expression mapping (eQTL) analysis used the same method described above for GWAS. The association analysis between the genome-wide SNPs and the identified candidate gene expression level was performed.

### 2.4 Data analysis

The line mean-based broad-sense heritability (*H^2^*) for each trait was calculated as *H^2^* =*σ2 g*/(*σ2 g*+*σ2 e/n*), where *σ2 g* is genetic variance, *σ2 e* is error variance, and *n* is the number of environments. The estimates of *σ2 g* and *σ2 e* were obtained by the mixed linear model, treating genotype and environment as random effects (R Core Team, 2012). For each metabolite, the BLUP value for each line across environments was used to reduce environmental noise based on the mixed linear model implemented in the R package ‘LME4’ (R Core Team, 2012). The Pearson correlation between different phenolamides and between phenolamides and other agronomic traits of this association panel (Yang et al., 2014) were calculated in subpopulations using the R function COR. TEST (www.r-project.org). Cytoscape v3.9.1 (http://www.cytoscape.org/download.php) was used for visualization.

### 2.5 Expression profiling of candidate genes and protein–protein interaction network

The expression profiling of candidate genes was analyzed through the transcriptomic data of the B73 maize inbred line in different seed development stages, including 0 days after pollination (S0), S2, S3, S4, S6, S8, S10, S12, S14, S16, S18, S20, S22, S24, S26, S28, S30, S32, S34, S36 and S38 (Chen et al., 2014a). The chiplot (https://www.chiplot.online/) was used to visualize the expression profiling of candidate genes based on the defaults options.

Protein-protein interaction network analysis was performed using the STRING database defaults options (https://string-db.org/) based on confirmed and predicted interactions. The interaction network was visualized by Cytoscape v3.9.1 (http://www.cytoscape.org/download.php). The network nodes represent proteins, and the edges represent protein-protein interactions. The GO (Gene Ontology) analysis of interaction proteins was performed using AgriGO v2.0 (http://systemsbiology.cau.edu.cn/agriGOv2/), and the analysis results (*p*<0.01) were imported into the online tool REVIGO (http://revigo.irb.hr/) and then visualized using software chiplot.

## 3 Results

### 3.1 Natural variations in phenolamides in maize kernels

Using high-throughput liquid chromatography tandem mass spectrometry (LC–MS/MS), we assessed the variation in phenolamides content in dry matured maize kernels, which included two recombinant inbred line populations (RIL), B73/BY804 (BB) and ZONG3/YU87-1 (ZY), and an association panel (AMP) harvested from multiple environments (simply called AMPE1, AMPE2 for AMP, BBE1, BBE2 for BB RIL population, and ZYE1 and ZYE2 for ZY RIL population, which are described in detail in “Materials and Methods”). In a previous study, 748 and 735 metabolites were detected in AMPE1 and AMPE2, respectively (Wen et al., 2014), and the chemical structures of 184 metabolites were identified or annotated in BB and ZY RIL populations (Wen et al., 2015). In the current study, we extract the profile of phenolamides from these previous datasets, which includes 16 phenolamides. Among them, 16, 16, 15, 16, 15, and 16 phenolamides were found in AMPE1, AMPE2, BBE1, BBE2, ZYE1, and ZYE2, respectively, and 15 phenolamides were detected in all six environments (**Table 1**).

**Table 1:** Detailed information of 16 phenolamides detected in this study.

No.	Peak no.	Level	Ret. Time (min)*	Putative phenolamides name	AMPE1	AMPE2	BBE1	BBE2	ZYE1	ZYE2
1	n0130	C	4.8	N-Feruloylputrescine	√	√	√	√	√	√
2	n0183	B	6.45	N-(coumaroyl-O-hexoside)-spermidine	√	√	√	√	√	√
3	n0271	C	9.63	Dicoumaroylputrescine	√	√	√	√	√	√
4	n0380	B	5.64	N-(caffeoyl-O-hexoside)-spermidine	√	√	√	√	√	√
5	n0381-1	B	9.9	Diferuloylputrescine	√	√	√	√	√	√
6	n0412	C	6.24	N-(feruloyl-O-hexoside)-spermidine	√	√	√	√	√	√
7	n0436	C	6.73	N1, N10-Diferuloylspermidine	√	√	√	√	√	√
8	n0439	B	10.2	N’,N’’-Feruloyl,caffeoyl-spermidine derivative	√	√	√	√	√	√
9	n0945	C	6.59	N-Coumaroylputrescine	√	√	√	√	√	√
10	n0979	B	5.08	N-Coumaroylagmatine	√	√	√	√	√	√
11	n1048	B	4.63	N-Coumaroyl-spermidine derivative	√	√	×	√	×	√
12	n1243	C	10.8	N,N-Di-coumaroyl-N-feruloylspermidine	√	√	√	√	√	√
13	n1376	B	5.33	Feruloylagmatine derivative	√	√	√	√	√	√
14	n1377	C	7.69	N-Feruloylagmatine	√	√	√	√	√	√
15	n1394	C	5.52	N-Feruloyl, N-methoxyagmatine	√	√	√	√	√	√
16	n1544-1	B	11.3	N,N-caffeoyl, Feruloyl-spermidine derivative	√	√	√	√	√	√

*Ret. Time, Retention time, in minutes (difference in Ret.Time between ES(+) and ES(-) modes was less than XX minutes), Identification level (B; C)- (B) MS/MS; (C) MSE.

**Figure 1:**
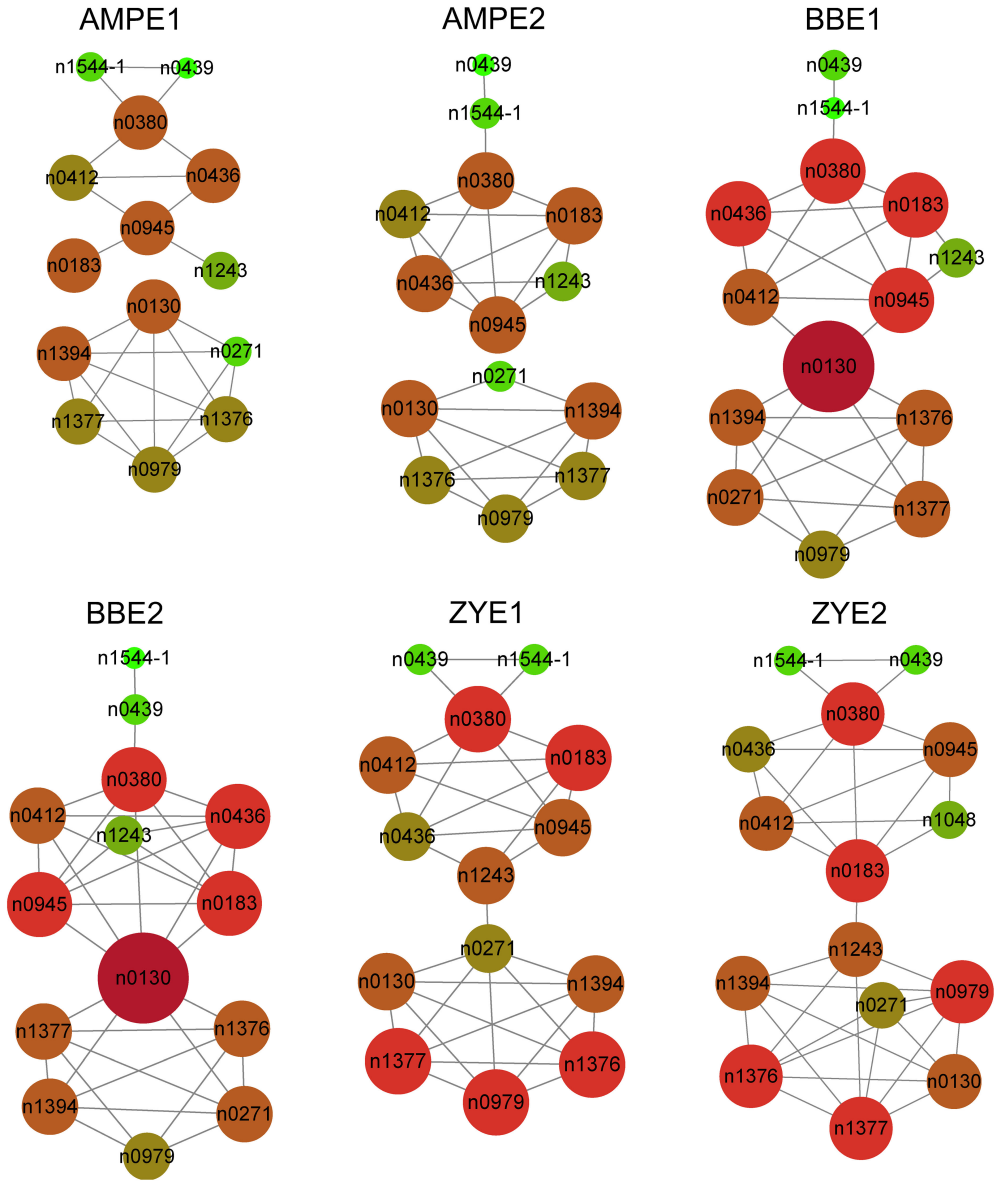
Correlation coefficient based network of all phenolamides in each experiment for AMP and both BB and ZY populations. |r| ≥ 0.3 for correlation coefficient between two phenolamides was used to construct the network.

The phenolamides levels varied widely in AMPE1, AMPE2, BBE1, BBE2, ZYE1, and ZYE2 (**Supplementary Tables 1**, **2**). Variation ranged from a 5.2-fold difference in N-(coumaroyl-O-hexoside)-spermidine to a 40663.0-fold difference in the N-coumaroyl-spermidine derivative and a 4.3-fold difference in N-(coumaroyl-O-hexoside)-spermidine to an 83045.1-fold difference in the N’,N’’-feruloyl, caffeoyl-spermidine derivative in association and linkage mapping populations, respectively (**Supplementary Tables 1**, **2**). The skewness, kurtosis and other detailed information for each phenolamide are shown in **Supplementary Tables 1**, **2**. In AMP, all phenolamides have broad-sense heritability (*H^2^*) greater than 0.4, and over 87.5% of phenolamides have *H^2^* greater than 0.6. Over 86.7% and 57.1% of phenolamides had *H^2^* greater than 0.5 in the BB and ZY populations, respectively (**Supplementary Table 3**, **Supplementary Figure 1**). We constructed correlation coefficient networks based on phenolamides levels detected in each experiment with R > 0.3. We found more intense interactions among n1544-1 (N,N-caffeoyl, feruloyl-spermidine derivative), n0439 (N’,N’’-feruloyl, caffeoyl-spermidine derivative), n0380 (N-(caffeoyl-O-hexoside)-spermidine), n0412 (N-(feruloyl-O-hexoside)-spermidine), n0436 (the N1, N10-diferuloylspermidine), n0945 (N-coumaroylputrescine), n0183 (N-(coumaroyl-O-hexoside)-spermidine) and n1243 (N,N-di-coumaroyl-N-feruloylspermidine). n0130 (N-Feruloylputrescine), n1377 (N-Feruloylagmatine), n1376 (Feruloylagmatine derivative), n1394 (N-Feruloyl, N-methoxyagmatine), n0271 (Dicoumaroylputrescine) and n0979 (N-Coumaroylagmatine) showed more intense interactions as well (**Figure 1**, **Supplementary Table 4**).

### 3.2 Correlation analysis with other traits

Phenolamides are an important class of metabolites in maize kernels. To explore how the kernel phenolamides are coordinated with other phenotypic traits. Pearson correlation coefficients were calculated using the COR. test in R to detect the statistical correlations between kernel phenolamides content and 23 other phenotypic traits previously measured in the same association panel. These 23 phenotypic traits included the morphological attributes plant height (PH), ear height (EH), ear leaf width and length (LW, LL), tassel main axis length (TML), tassel branch number (TBN), and leaf number above ear (ULN); yield-related traits ear length and diameter (EL, EW), cob diameter (CD), kernel number per row (RKN), row number per ear (ERN), hundred kernel weight (HW), cob weight (CW), kernel width (KW), kernel length (KL) kernel thickness (KT); maturity traits days to heading, anthesis, and silking (HD, PS, ST); disease resistance maize rough dwarf virus (MRDV) and sugarcane mosaic virus (SCMV); and cob color (CC).

**Figure 2:**
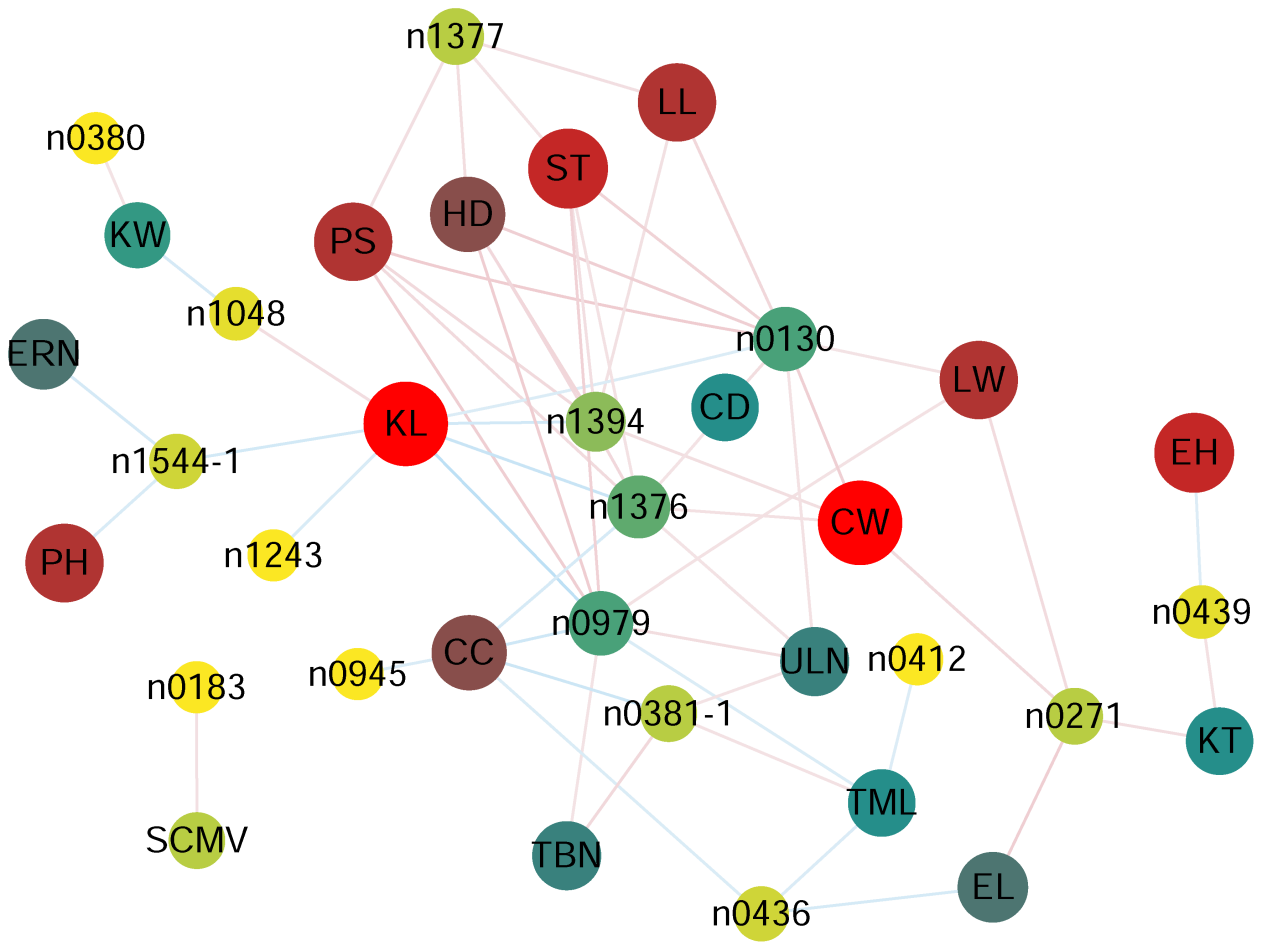
Correlation coefficient based network between phenolamides and agronomy traits in each experiment for AMP populations. *p* < 0.05 between phenolamides agronomy traits and was used to construct the network. CC, cob color, CD, cob diameter, CW, cob weight, PH, plant height, EH, ear height, LL, ear leaf length, LW, ear leaf width, EL, ear length, TML, tassel main axis length, TBN, tassel branch number, ULN, up leaf number, ERN, ear row number, KW, kernel width, KL, kernel length, KT, kernel thick, HD, Headingdate, PS, pollenshed, ST, silkingtime, SCMV, sugarcane mosaic virus.

The results showed that the 16 phenolamides were all significantly correlated (*p* < 0.05) with one or several of 19 phenotypic traits except EW, RKN, HW and MRDV. The content of n0130 (N-feruloylputrescine), n1377 (N-feruloylagmatine), n1376 (feruloylagmatine derivative), n1394 (N-feruloyl, N-methoxyagmatine) and n0979 (N-coumaroylagmatine) exhibited significant HD, PS and ST (**Figure 2**, **Supplementary Table 5**), suggesting that increasing the content of N-feruloylputrescine, N-feruloylagmatine, feruloylagmatine derivative, N-feruloyl, N-methoxyagmatine and N-coumaroylagmatine might increase days to heading, anthesis, and silking. N1048 (N-coumaroyl-spermidine derivative) showed a significant positive correlation (*p* = 0.0117; R = 0.144) with KL and a significant negative correlation (*p* = 0.0111; R = -0.145) with KW. N0183 (N-(coumaroyl-O-hexoside)-spermidine) showed a significant positive correlation (*p* = 0.0217; R = 0.131) with sugarcane mosaic virus (**Figure 2**, **Supplementary Table 5**), implying that increasing the N-coumaroyl-spermidine derivative content might increase the resistance of sugarcane mosaic virus. The content of N,N-caffeoyl, feruloyl-spermidine derivative was significantly negatively correlated with KL (*p* = 6.86×10^-3^; R = -0.154), ERN (*p* = 9.74×10^-3^; R = -0.147), and PH (*p* = 0.0199; R = -0.133), suggesting that an increase in N,N-caffeoyl, feruloyl-spermidine derivative content may decrease the KL, ERN and PH (**Figure 2**, **Supplementary Table 5**). More detailed information on the correlation is shown in **Figure 2** and **Supplementary Table 5**.

### 3.3 Linkage mapping for phenolamides levels in the two RIL populations

Two RIL populations (BB and ZY) were genotyped with a high-density SNP array (Pan et al., 2016) and were used for QTL mapping for phenolamides. For the BB population, 58 and 58 QTLs were mapped for 15 and 16 traits in BBE1 and BBE2, respectively, with an average of 3.9 and 3.6 QTLs per trait, respectively (**Table 2**, **Supplementary Table 6**). Only nine QTLs were detected for the six common phenolamides in BBE1 and BBE2 (**Supplementary Table 6**). Each QTL explained 4.79%-20.20% (BBE1) and 2.75%-76.38% (BBE2) of the phenotypic variation, with averages of 9.47% and 10.78% (**Table 1**, **Supplementary Table 6**), respectively. Forty-one QTLs that explained greater than 10% of the phenotypic variation (R^2^ = 10.41%-76.38%) were identified in two experiments.

**Table 2:** Summary of significant loci-trait associations identified by GWAS and QTL identified by linkage mapping.

	AMPE1	AMPE2	BBE1	BBE2	ZYE1	ZYE2
Number of traits	12/16	13/16	15/15	16/16	11/15	16/16
Number of loci	38	35	58	58	39	67
Average loci per trait	3.2 ± 2.6	2.7 ± 1.9	3.9 ± 2.6	3.6 ± 1.5	3.6 ± 2.0	4.2 ± 1.8
Average PVE per loci (%)	10.07 ± 4.40	10.01 ± 2.99	9.47 ± 3.60	10.78 ± 9.18	9.51 ± 2.70	11.40 ± 5.22

For the ZY RIL population, 39 and 67 QTLs were detected for 11 and 16 phenolamides traits in ZYE1 and ZYE2, respectively, with averages of 3.6 and 4.2 QTLs, respectively (**Table 2**, **Supplementary Table 6**). Only four QTLs were detected for the three common phenolamides in ZYE1 and ZYE2 (**Supplementary Table 6**). Each QTL explained 6.59%-17.54% (ZYE1) and 5.73%-29.98% (ZYE2) of the phenotypic variation, with averages of 9.51% and 11.40% (**Table 1**, **Supplementary Table 6**), respectively. Forty-three QTLs that explained greater than 10% of the phenotypic variation (R^2 =^ 10.02%-29.98%) were identified in two experiments (**Supplementary Table 6**). For the same trait, only 15 QTLs were detected in more than one population, implying that different low-frequency QTLs existed in different genetic backgrounds. We analyzed the resolution of QTL mapping, and the results showed a 19.82% (44/222) QTL interval less than 1 Mb and a 67.57% (150/222) QTL interval less than 5 Mb (**Supplementary Figure 2**, **Supplementary Table 6**). There are many QTLs explaining more than 10%, but only 2 and 6 more than 20% (for BB and ZY respectively).

### 3.4 GWAS for phenolamides levels

GWAS was performed using an association panel including 368 maize diverse inbred lines (Wen et al., 2014) and 1.25 million high-quality single nucleotide polymorphisms (SNPs) with minor allele frequency (MAF) >0.05 (Fu et al., 2013; Liu et al., 2017). A total of 73 loci were identified by GWAS at a significance level of *p* ≤ 2.04 × 10^−6^ in two experiments (AMPE1, AMPE2) (**Table 2**). Briefly, 38 and 35 loci were identified for 12 phenolamides in AMPE1 and 13 phenolamides in AMPE2, with an average of 3.2 and 2.7 loci for each trait, respectively, and only five of these loci were conserved for the same phenolamides in both experiments. Each locus could explain phenotypic variation (R^2^) ranging from 6.97% to 23.10% and 7.10% to 17.71%, with means of 10.07% and 10.01%, respectively. Twenty-four loci with effects greater than 10% were identified in two environments (**Supplementary Table 7**). Detailed information on the GWAS results, including the *p* value and R^2^ of each locus, physical position and minor allele frequency (MAF) of the lead SNP, annotation, eQTL, and correlation between phenotype and expression of the most likely candidate gene, is provided in **Supplementary Table 7**.

**Figure 3:**
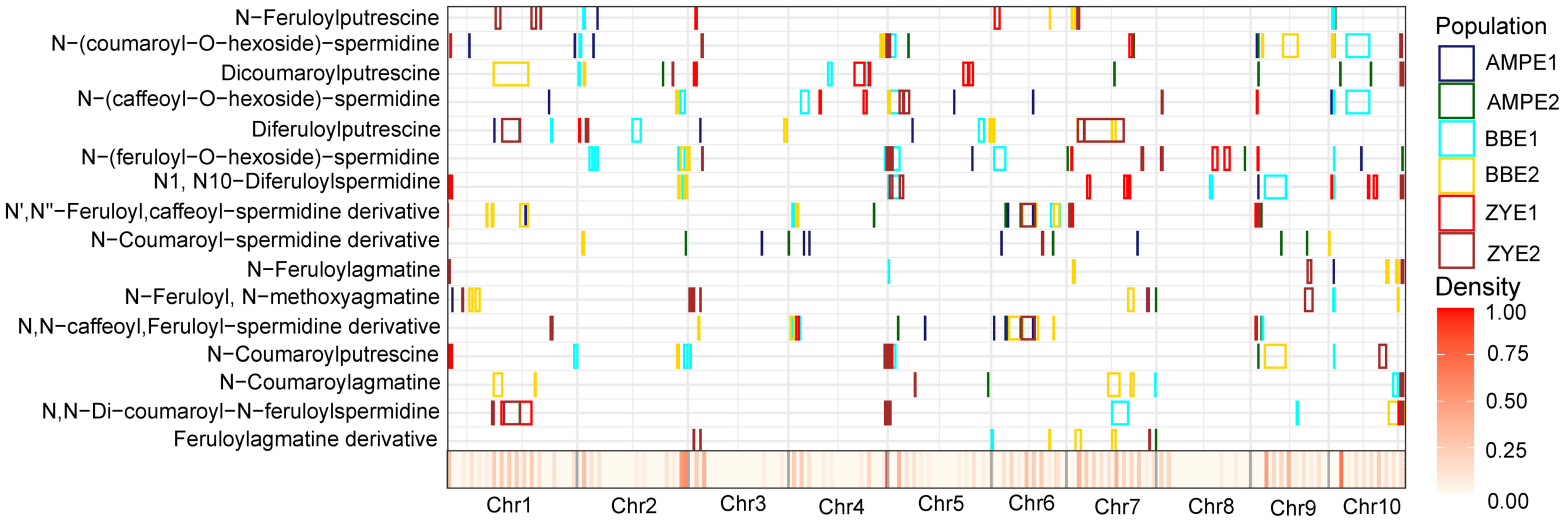
Chromosomal distribution of phenolamides loci and QTLs identified in this study. QTL regions (represented by the confidence interval for linkage mapping and the 100kb up- and downstream of the lead SNP for association mapping) across the maize genome responsible for phenolamides levels from the different populations are shown as midnight blue (AMPE1), green (AMPE2), cyan (BBE1), yellow (BBE2), red (ZYE1) and brown (ZYE2) boxes, respectively. The x axis indicates the genetic positions across the maize genome in Mb. Heatmap under the x axis illustrates the density of amino acid loci and QTLs across the genome.

The colocalization of QTLs and/or significant loci for the same trait identified across different environments or different populations is summarized. In total, 10 trait-loci combinations that are 8 QTLs corresponding to five traits were detected in more than one environment or population (AMP, BBRIL, ZYRIL) in this study (**Figure 3**, **Supplementary Table 6**, **7**). Detailed analyses of the candidate genes underlying these loci will provide useful further information concerning the phenolamides biosynthetic pathway.

### 3.5 Prediction and annotation of candidate genes

**Figure 4:**
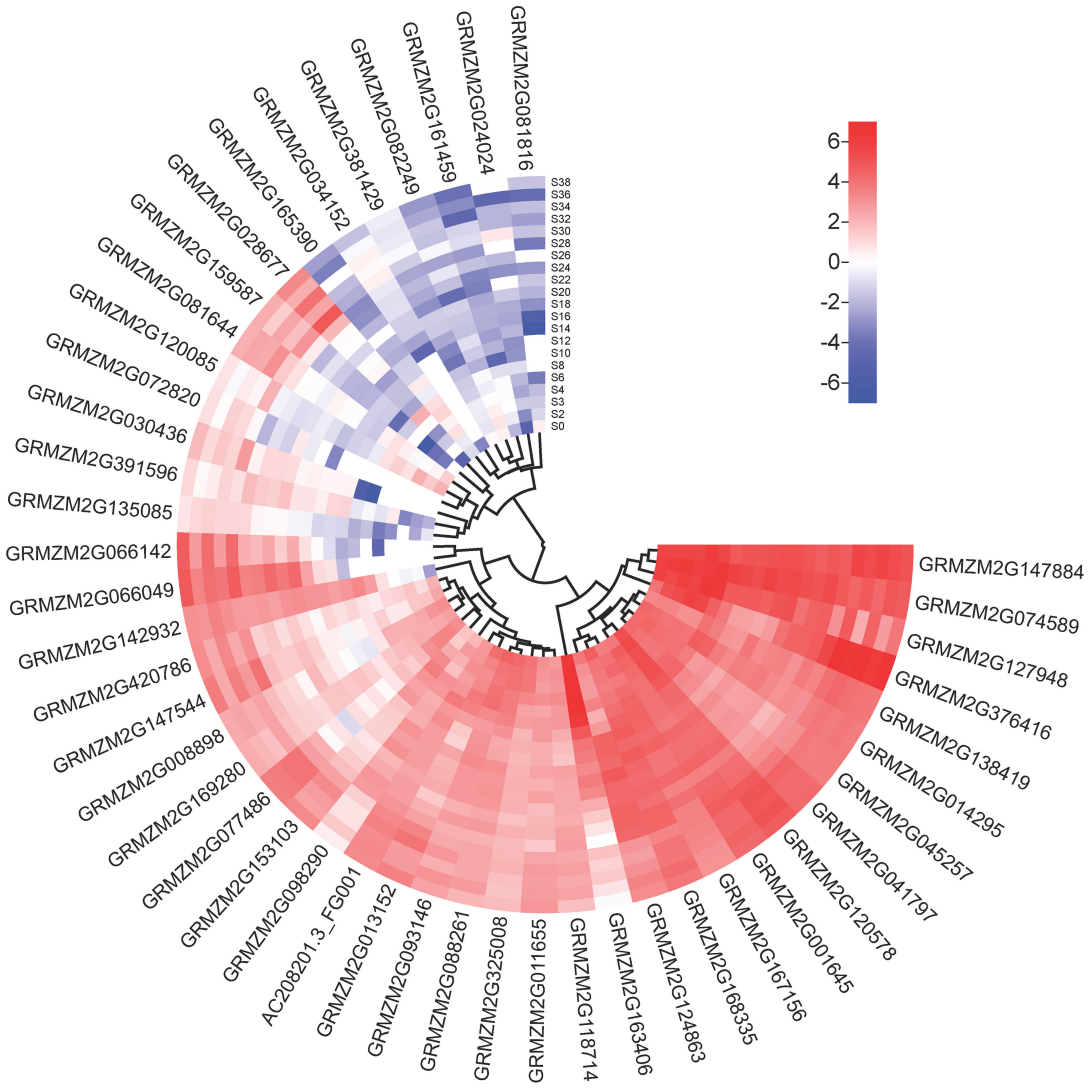
Heatmap of the expression profiles of candidate genes. The scale bars represent standardized gene levels. S0-S38 indicate days after pollination of maize seed.

Subsequently, limited overlaps were found between the loci (10/73) identified by GWAS and the QTLs identified by linkage mapping for the same trait in the present study. A total of 58 unique candidate genes corresponding to 73 trait-locus associations identified in two experiments were annotated, and other potential candidate genes within 200 kb (100 kb upstream and downstream of the lead SNPs) of the 73 loci are also listed in **Supplementary Table 7**. Among these candidate genes, 46 genes that may affect phenolamides were found in different developmental stages of maize kernel (**Figure 4**). Based on the current database, among the 58 candidate genes, those encoding enzymes involved in amine and derivative metabolic processes accounted for 31%, the enzymes involved in other biological processes accounted for 19%, transcription factors accounted for 10%, and the unknown functions accounted for 17% (**Supplementary Figure 3**).

Expression QTLs (eQTL, n = 368) were identified for a plurality of these candidate genes (55.2%, or 32/58) using the previous RNA-sequencing data of immature kernels (Fu et al., 2013). Significant correlations (*p* < 0.05, n = 335-339) between the expression level of the candidate genes with eQTLs identified and the phenotypic variation of the corresponding phenolamides were found in 14 cases (24.1%) (**Supplementary Table 7**), which suggests that some of these loci affect phenotypic variation via transcriptional regulation.

### 3.6 Protein–protein interaction network analysis

Proteins usually regulate the growth and development of plants in complex interrelated networks. To understand the metabolism of phenolamide-related traits in maize, protein-protein interaction networks were constructed of 46 highly expressed candidate genes through the STRING database (https://string-db.org/). Then, 358 genes were detected that were associated with 43 candidate genes (**Supplementary Figure 4**, **Supplementary Table 8**). Gene Ontology (GO) term analysis of the protein-protein interrelated genes revealed significant enrichment in terms relating to cellular nitrogen metabolism, amine metabolism, amino acid and derivative metabolism, organic acids and other processes (**Supplementary Figure 5**).

### 3.7 Analysis of candidate genes

**Figure 5:**
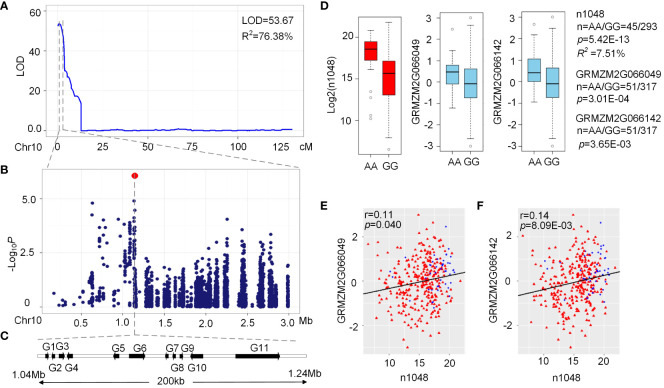
Validation of association analysis using QTL Interval. **(A)** LOD curves of QTL mapping for level of n1048 in maize kernels on chromosome 10. **(B)** Scatterplot of association results between SNPs in the confidence interval and the level of n1048. Association analysis was performed using the mixed linear model controlling for the population structure (Q) and kinship (K). **(C)** The candidate genes of 200kb in the confidence interval. **(D)** Box plot for n1048 (red) from AMPE1 and expression of GRMZM2G066142 (sky blue) and GRMZM2G066049 (sky blue). **(E)** Plot of correlation between the n1048 level in AMPE1 and the normalized expression level of the GRMZM2G066142 and GRMZM2G066049, red triangle and blue spot represented the lead SNP GG and AA, respectively. The r value is based on a Pearson correlation coefficient. The *p* value is calculated using the Student’s-t test.

A major QTL on chromosome 10 (LOD = 53.67, R^2 =^ 76.38%) affecting n1048 (N-coumaroyl-spermidine derivative) was identified in the BBE2 RIL population (**Figure 5A**) with a confidence interval of just 2.1 cM (0-2.1 cM) and a physical length of 2.04 Mb (0-2.04 Mb) (**Supplementary Table 6**). A GWAS signal was detected within the QTL interval located at 1.14 Mb (*p* = 8.46×10^-7^, n = 339, **Figure 5B**). Eleven candidate genes were obtained within the 200 kb region around the peak, including two *ZmACTs* (GRMZM2G066142 and GRMZM2G066049), three transposable elements (GRMZM2G365485, GRMZM5G854762 and GRMZM2G057831), and six unknown genes (**Figure 5C**). The two *ZmACT* (GRMZM2G066142 and GRMZM2G066049) genes are located approximately 55-60 kb upstream of the lead SNP chr10. S_1144300 (**Figures 5B, C**). *ZmACT* encodes agmatine coumaroyltransferase, which catalyzes the production of hydroxycinnamoyl derivatives such as p-coumaroylagmatine, p-coumaroylputrescine, feruloylagmatine and feruloylputrescine (Muroi et al., 2009). The lead SNP was strongly associated with GRMZM2G066142 (*p* = 3.65 × 10^−3^, n = 368) and GRMZM2G066049 expression levels (*p* = 3.01 × 10^−3^, n = 368) (**Figure 5E**) and phenotypic traits from AMPE1 (*p* = 5.42 × 10^−13^, R^2^ = 7.51%, n = 338) and AMPE2 (*p* = 1.49 × 10^−13^, R^2^ = 8.43%, n = 335), respectively (**Figure 5D**). Subsequently, the expression levels of GRMZM2G066049 were significantly positively correlated with the level of n1048 from AMPE1 (*p* = 0.040, r = 0.11, n = 339). The expression levels of GRMZM2G066142 were significantly positively correlated with the level of n1048 from AMPE1 (*p* = 8.09 × 10^−3^, r = 0.14, n = 339) (**Figure 5F**). These results imply that these two genes are candidate genes.

A strong signal (*p* = 4.49 × 10^−7^, n = 339) was identified on chromosome 9 (**Supplementary Table 7**), associated with n0183 (N-(coumaroyl-O-hexoside)-spermidine) from AMPE1, which could explain 7.89% of the phenotypic variation. The *Bz1* (GRMZM2G165390) gene is located approximately 153 kb upstream of the lead SNP chr9. S_11929632 (**Supplementary Table 7**). *Bz1* encodes anthocyanin-3-O-glucosyltransferase, a key enzyme in the anthocyanin synthesis pathway. The lead SNP was strongly associated with the *Bz1* expression level (*p* = 1.59 × 10^−17^, n = 367) and phenotypic trait (*p* = 1.26 × 10^−6^, R^2^ = 7.89%, n = 338). Subsequently, a strong cis-eQTL was detected for *Bz1* (*p* = 1.98 × 10^−20^, n = 368, MLM, **Supplementary Figure 6**), and the expression level of *Bz1* was significantly negatively correlated with the level of n0183 (r = -0.16, *p* = 2.75 × 10^−3^, n = 338, **Supplementary Figure 6**, **Supplementary Table 7**).

The significant SNP chr1. S_140716298 (in the 3′UTR of GRMZM2G159587) was significantly associated with n0381-1 (diferuloylputrescine) (*p* = 1.39 × 10^−11^, n = 274) and n1243 (N,N-di-coumaroyl-N-feruloylspermidine) (*p* = 9.06 × 10^−7^, n = 274) from AMPE2, which accounted for 16.94% and 9.04% of the phenotypic variance n0381-1 and n1243, respectively (**Supplementary Figure 7**, **Supplementary Table 7**). *ZmGR* (GRMZM2G159587) encodes glyoxylate reductase. The lead SNP was strongly associated with the *ZmGR* expression level (*p* = 8.81 × 10^−4^, n = 300). Subsequently, a strong cis-eQTL was detected for *ZmGR* (*p* = 1.97 × 10^−9^, n = 368, MLM, **Supplementary Figure 7**), and the expression level of *ZmGR* was significantly positively correlated with the levels of n0381-1 (r = 0.14, *p* = 9.40 × 10^−3^, n = 274) and n1243 (r = 0.15, *p* = 6.07 × 10^−3^, n = 274, **Supplementary Figure 7**, **Supplementary Table 7**).

## 4 Discussion

Phenolamides are important secondary metabolites in plant species. They are important for defense responses against pathogens, insect herbivores, sulfur starvation, salt stress, protection against UV irradiation and floral induction and development (Demkura et al., 2010; Onkokesung et al., 2012; Gaquerel et al., 2013; Figon et al., 2021; Fang et al., 2022; Xu et al., 2022). Overexpression of endogenous tyramine hydroxycinnamoyltransferase increased its resistance to *Pseudomonas syringae* in tomato (Campos et al., 2014), the ectopic expression of the *AtACT* in torenia plants rendered them more resistant to Botrytis cinerea (Muroi et al., 2012). Meanwhile, the accumulation of p-coumaroylagmatine, p-coumaroylputrescine, and caffeoylputrescine reduced spore germination of *P. infestans* on the potato leaf surface (Dobritzsch et al., 2016). Through jasmonate-mediated activation of defense-related genes and accumulation of aromatic phenolamides in Qingke increased the resistance to powdery mildew (Xu et al., 2022). In this study, we found that the n0183 (N-(coumaroyl-O-hexoside)-spermidine) showed a significant positive correlation (*p* = 0.0217; R = 0.131) with sugarcane mosaic virus, and this result showed that n0183 might increase resistance to sugarcane mosaic virus. In addition, the levels of n0130, n1377, n1376, n1394 and n0979 exhibited significant positive correlations with HD, PS and ST (**Figure 2**, **Supplementary Table 5**), suggesting that increasing their contents might increase days to heading, anthesis, and silking.

Linkage analysis is a classical method for dissecting the genetic basis that underlies quantitative traits. Fine mapping based on the primary mapping results remains a conventional strategy. With the rapid development of sequencing technology, we could obtain an increasing number of molecular markers. However, due to the limited combinations and the narrow genetic background of the parents, linkage mapping is usually not very effective for complex quantitative traits. GWAS is characterized by a high density of SNPs and a large population, which can effectively solve the problem of low diversity and detection rate, but a large number of false-positive results will confuse the truly relevant sites and reduce the detection ability (Zhang et al., 2022). Currently, as an efficient approach, the combination of the GWAS approach and linkage analysis can help us quickly identify candidate genes. To date, only a few studies have focused on the genetic architecture of maize kernel PAs (Wen et al., 2014).

In the present study, we focused on the phenolamides that were found in mature kernels harvested from an association panel and two RIL populations grown across multiple environments. GWAS and linkage mapping were used to dissect the genetic basis of PA content in mature maize kernels from the aforementioned populations. Combining linkage mapping and GWAS for 16 PA traits revealed 58, 58, 39 and 67 QTLs and 39 and 36 significant loci, respectively. Only a few QTLs (15/222) could be identified in multiple RIL populations, and only 10 trait-loci combinations that were 8 QTLs corresponding to five traits were detected in more than one environment or population (**Supplementary Tables 6**, **7**). Similar results have also been reported in other metabolite studies in maize (Wen et al., 2014; Deng et al., 2017, 2015, 2016). These results implied that QTLs affecting PA composition were genetic background dependent. In this study, 73 loci were detected in AMPE1 and AMPE2 with 1.25 million SNPs, and only 23/73 loci colocalized with 1.06 million high-quality SNPs identified in the corresponding environment in a previous report (Wen et al., 2014). Therefore, a high-density map increased the QTL detection power and resolution (Liu et al., 2017). A protein–protein network was constructed based on the genes identified by GWAS (**Supplementary Figure 4**), and the interacting proteins were found. These proteins are enriched in terms relating to cellular nitrogen metabolism, amine metabolism, amino acid and derivative metabolism, organic acids and other processes (**Supplementary Figure 5**). Further studies are needed to fully explore the genetic control of phenolamides biosynthetic pathways.

## 5 Conclusion

An association panel and two RIL populations were used to identify candidate genes for 16 phenolamide traits in multiple environments. A total of 58, 58, 39 and 67 QTLs, explaining 9.47%, 10.78%, 9.51% and 11.40% of the phenotypic variation for each QTL on average, were mapped in BBE1, BE2, ZYE1 and ZYE2, respectively. Thirty-nine and 36 significant loci, explaining 10.00% and 9.97% of the phenotypic variation for each locus on average, were identified in two different environments. GRMZM2G066142, GRMZM2G066049, GRMZM2G165390 and GRMZM2G159587 were further validated using bioinformatics approaches. These findings provide insights into the genetic basis of phenolamide biosynthesis in maize kernels, understanding phenolamide biosynthesis and its nutritional content and ability to withstand biotic and abiotic stress.

## Data availability statement

The original contributions presented in the study are included in the article/**Supplementary Material**. Further inquiries can be directed to the corresponding author.

## Author contributions

MD: Formal analysis, Investigation, Writing – original draft. QZ: Formal analysis, Investigation, Software, Writing – review & editing. SL: Formal analysis, Investigation, Software, Writing – review & editing. MJ: Data curation, Formal analysis, Investigation, Writing – review & editing. HL: Supervision, Writing – review & editing. JL: Funding acquisition, Supervision, Writing – review & editing.

## Supplementary material

Supplementary Figure 1 The *H^2^* distribution of phenolamides in different populations and environments.

Supplementary Figure 2 The QTL interval distribution of phenolamides in different RIL populations.

Supplementary Figure 3 Functional category annotations for 58 candidate genes and their respective percentages identified via GWAS as significantly associated with phenolamide traits in maize kernels.

Supplementary Figure 4 Protein-protein interaction networks of 43 expressed candidate genes. The node represents the protein, and the line represents interaction between the proteins. The red solid circles represent the proteins encoded by the candidate genes.

Supplementary Figure 5 Gene Ontology annotation of 401 interaction proteins from 43 GWAS candidate genes.

Supplementary Figure 6 GWAS for n0183 with significant SNP-trait association in this study. **(A)** Plot of the correlation between the n0183 level and the normalized expression level of *BZ1* (GRMZM2G165390). **(A)** Box plot for n0183 level (sky blue) and expression of *BZ1* (red). The r value is based on a Pearson correlation coefficient. The *p* value was calculated using Student’s t test.

Supplementary Figure 7 GWAS for n0381-1 and n1243 with significant SNP-trait association in this study. **(A)** Box plot for the n0381-1 level. **(B)** Box plot for the n1243 level. **(C)** Box plot for the expression of *ZmGR* (GRMZM2G159587). **(D)** Plot of the correlation between the n0381-1 level and the normalized expression level of *ZmGR* (sky blue). **(E)** Plot of the correlation between the n1243 level and the normalized expression level of *ZmGR* (sky blue). The r value is based on a Pearson correlation coefficient. The P value was calculated using Student’s t test.

Supplementary Table 1 Phenolamide intensities of each line in AMP and both BB and ZY populations planted across multiple environments.

Supplementary Table 2 Range and mean fold changes of phenolamide traits measured in AMP and both BB and ZY populations.

Supplementary Table 3 The heritability distribution of each phenolamide in different populations and environments.

Supplementary Table 4 Significant correlation between each phenolamide.

Supplementary Table 5 Significant correlation between phenolamides and agronomic traits.

Supplementary Table 6 QTL mapping summary of phenolamide-related traits detected from three RIL populations.

Supplementary Table 7 Significant loci associated with phenolamide traits identified by GWAS across two environments.

Supplementary Table 8 The list of significant Gene Ontology terms.
