# Supplementary figures and images for: Combining association with linkage mapping to dissect the phenolamides metabolism of the maize kernel
Source: Front Plant Sci. 2024 Apr 12;15:1376405. doi: 10.3389/fpls.2024.1376405 (PMC11047430; doi:10.3389/fpls.2024.1376405)

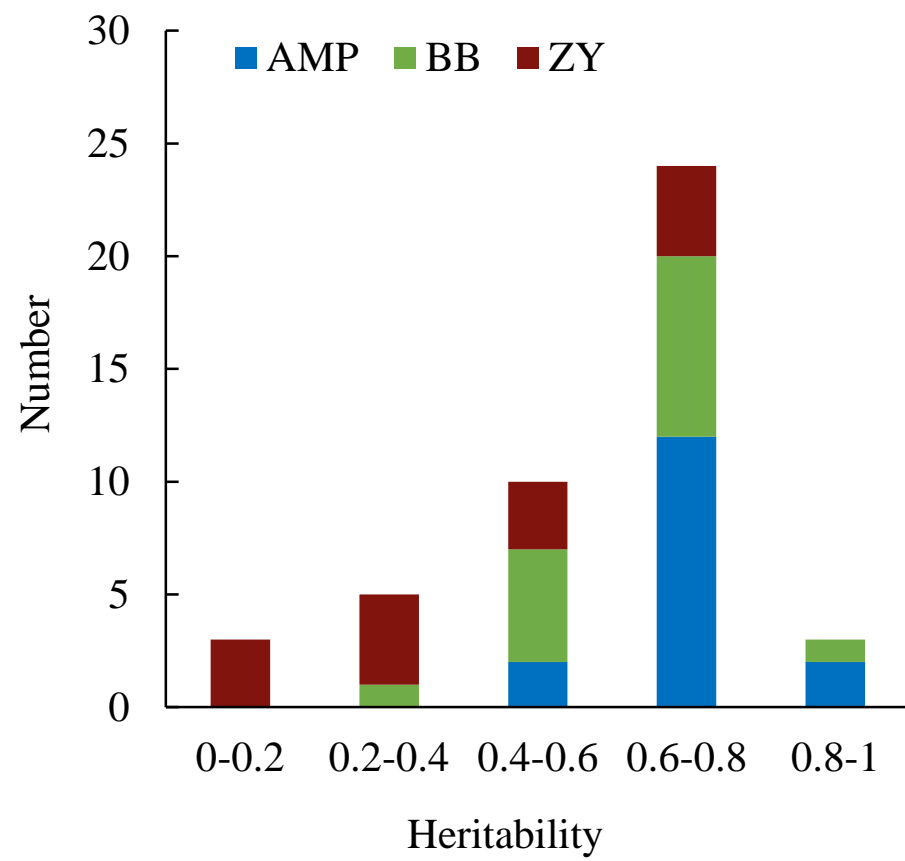

Figure S1

Supplement: Supplementary Figure 1 — The H2 distribution of phenolamides in different populations and environments. [file Image_1.pdf]

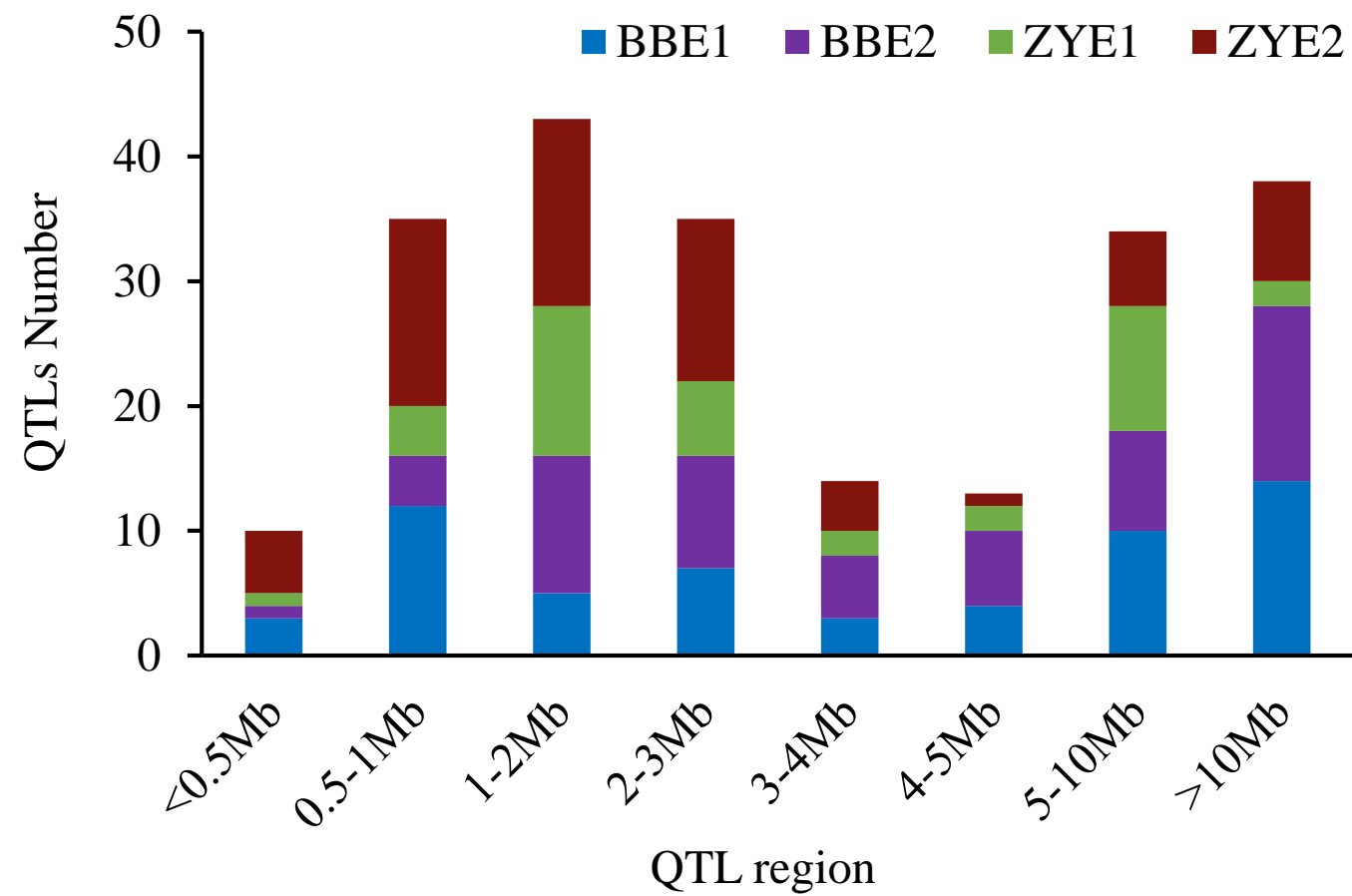

Figure S2

Supplement: Supplementary Figure 2 — The QTL interval distribution of phenolamides in different RIL populations. [file Image_2.pdf]

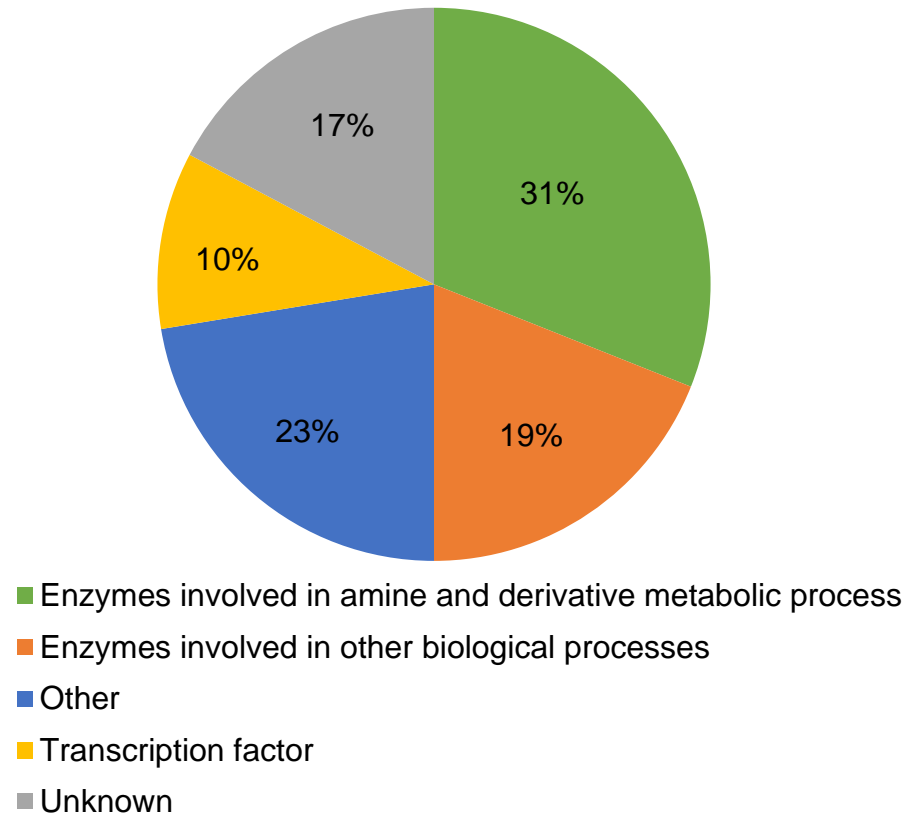

Figure S3

Supplement: Supplementary Figure 3 — Functional category annotations for 58 candidate genes and their respective percentages identified via GWAS as significantly associated with phenolamide traits in maize kernels. [file Image_3.pdf]

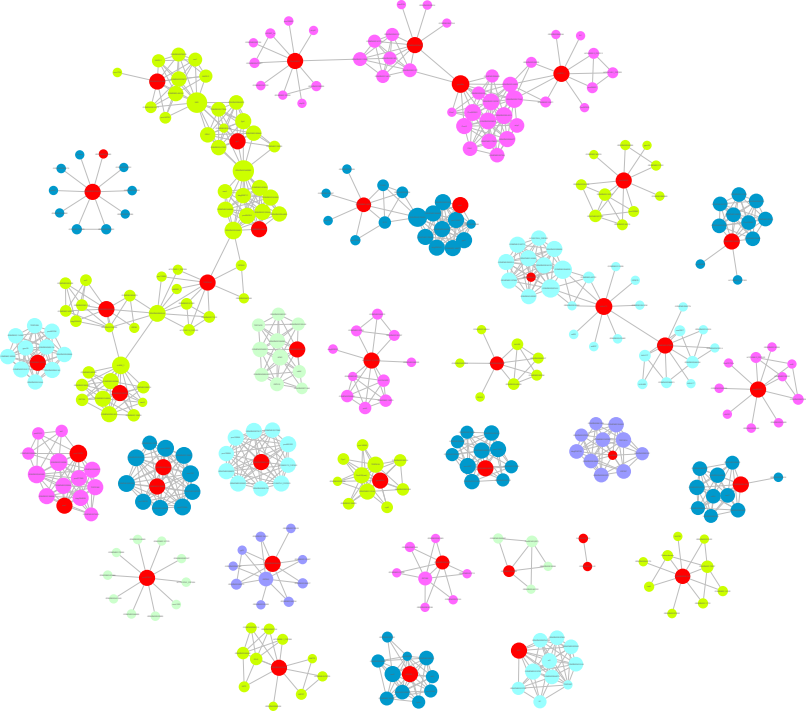

Supplement: Supplementary Figure 4 — Protein-protein interaction networks of 43 expressed candidate genes. The node represents the protein, and the line represents interaction between the proteins. The red solid circles represent the proteins encoded by the candidate genes. [file Image_4.pdf]

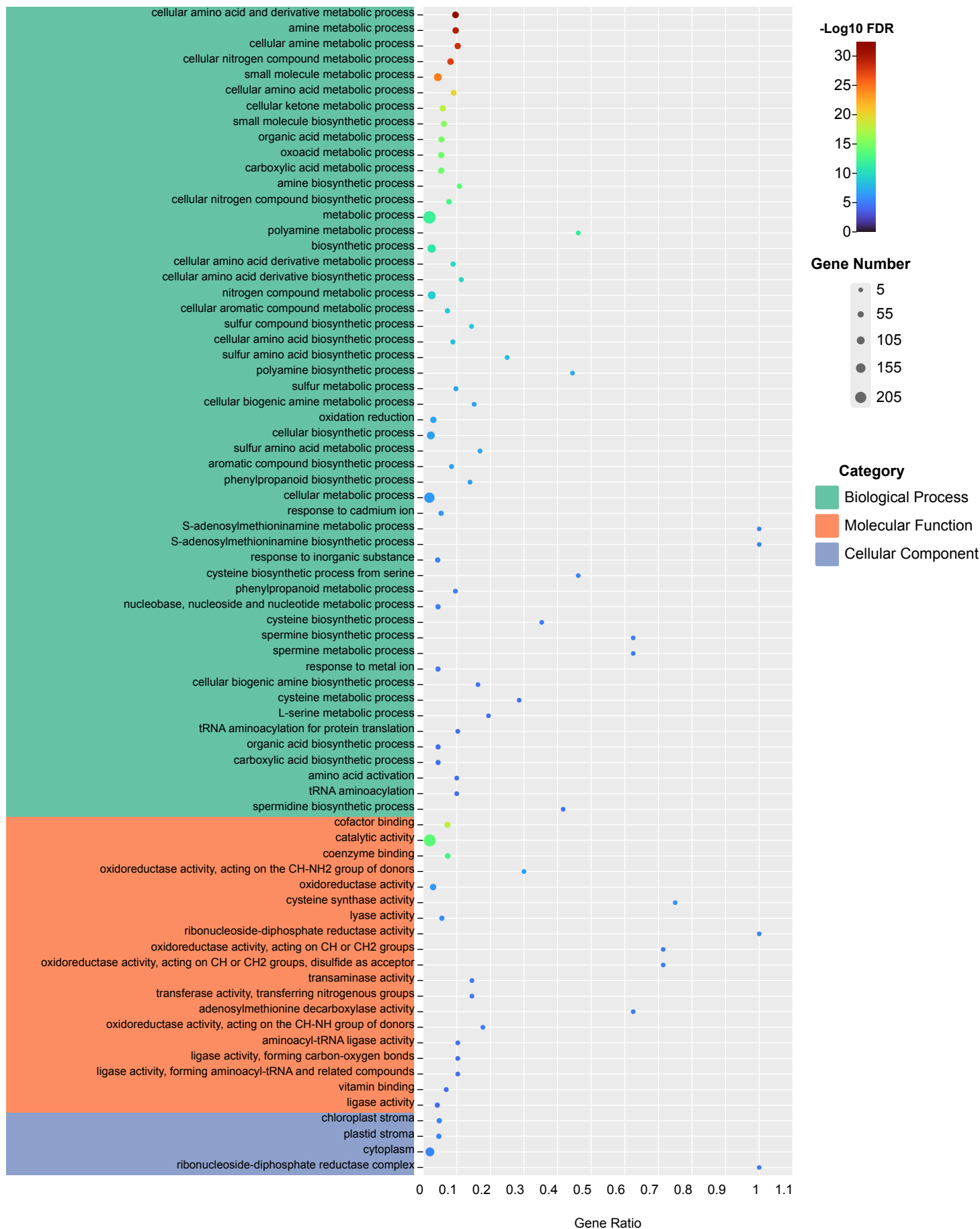

Supplement: Supplementary Figure 5 — Gene Ontology annotation of 401 interaction proteins from 43 GWAS candidate genes. [file Image_5.pdf]

**a**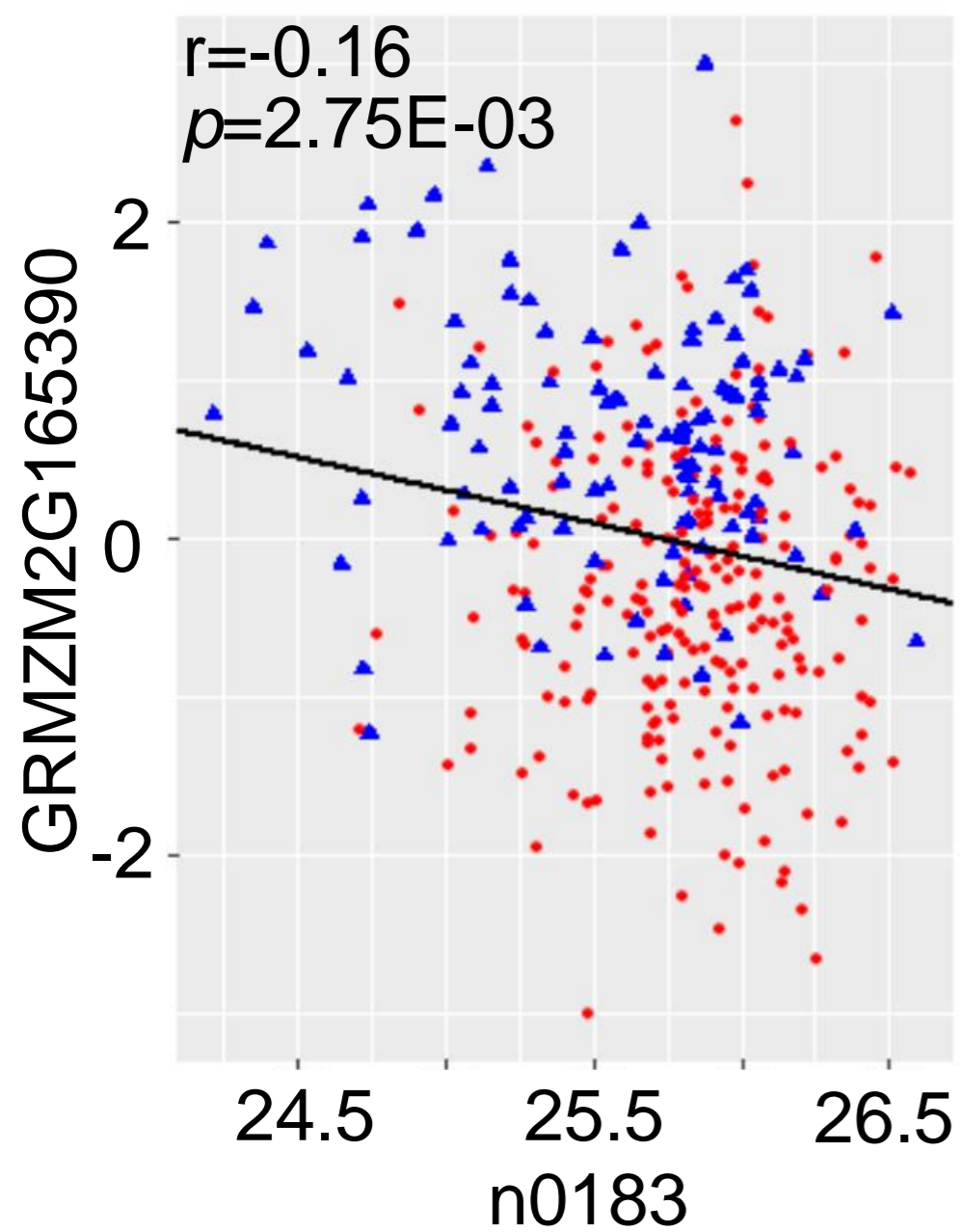**b**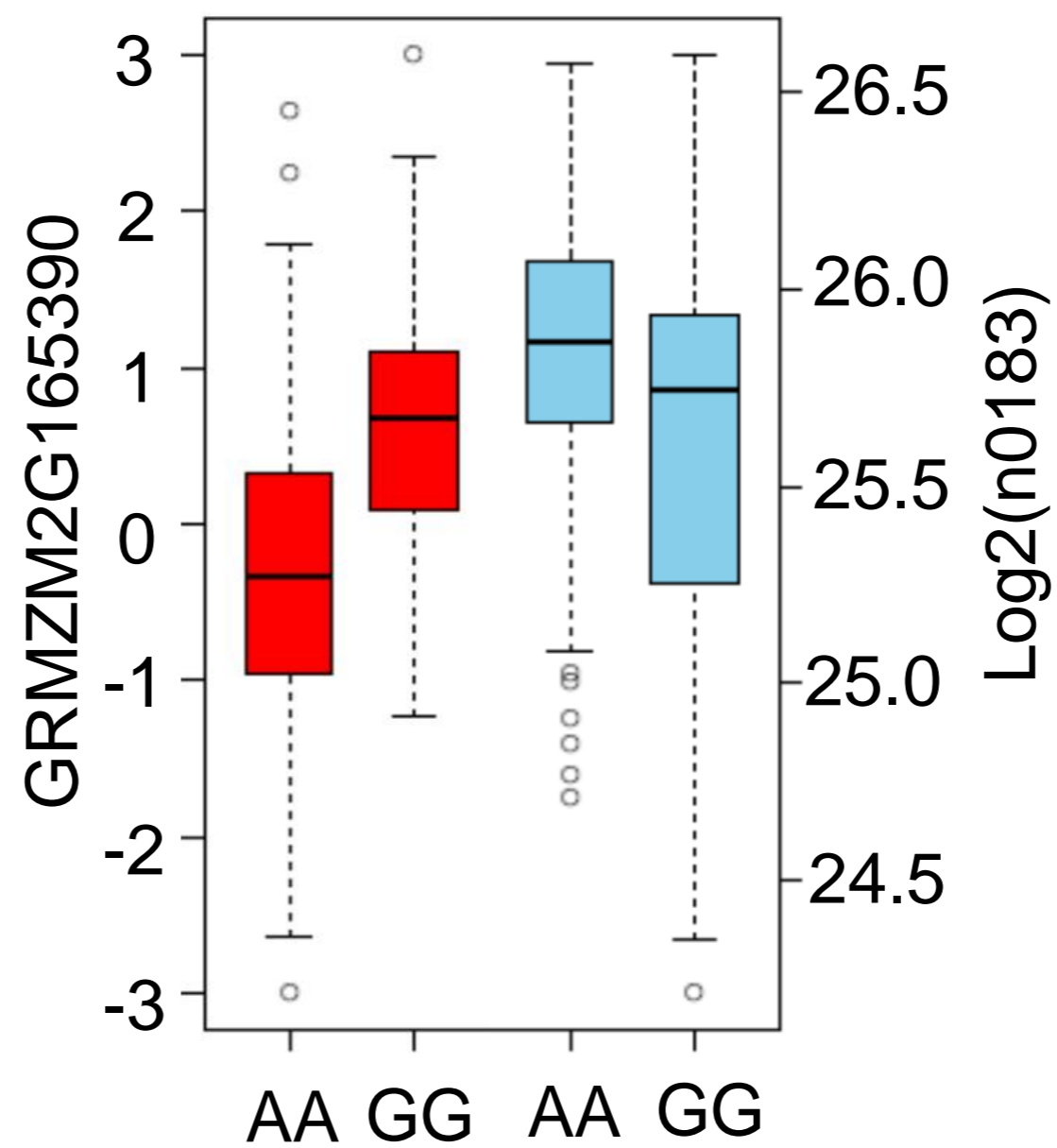

n0183  
n=AA/GG=228/110  
 $p = 1.26E-06$   
 $R^2 = 7.89\%$

GRMZM2G165390  
n=AA/GG=245/122  
 $p = 1.59E-17$

Supplement: Supplementary Figure 6 — GWAS for n0183 with significant SNP-trait association in this study. (A) Plot of the correlation between the n0183 level and the normalized expression level of BZ1 (GRMZM2G165390). (A) Box plot for n0183 level (sky blue) and expression of BZ1 (red). The r value is based on a Pearson correlation coefficient. The p value was calculated using Student’s t test. [file Image_6.pdf]

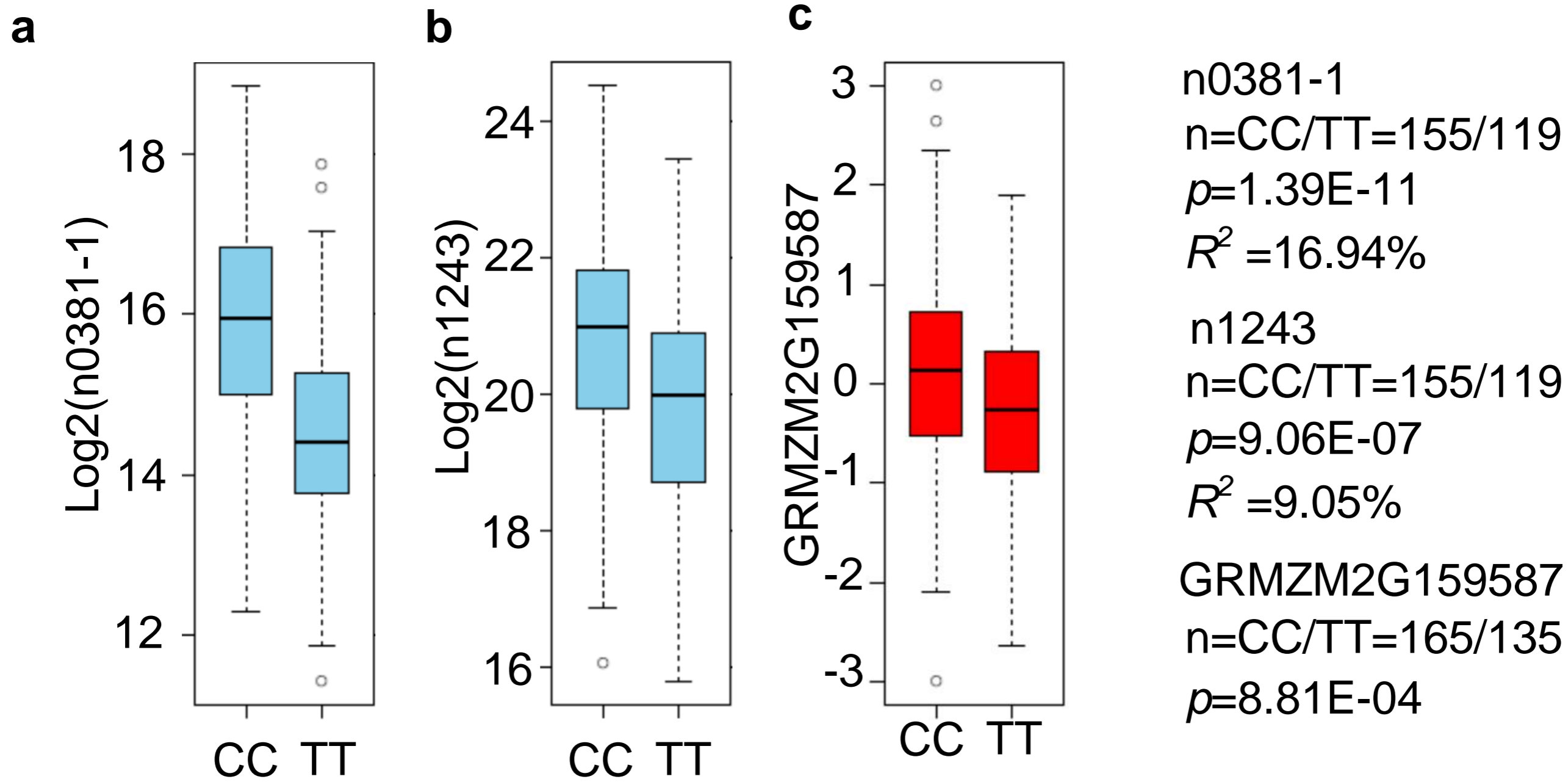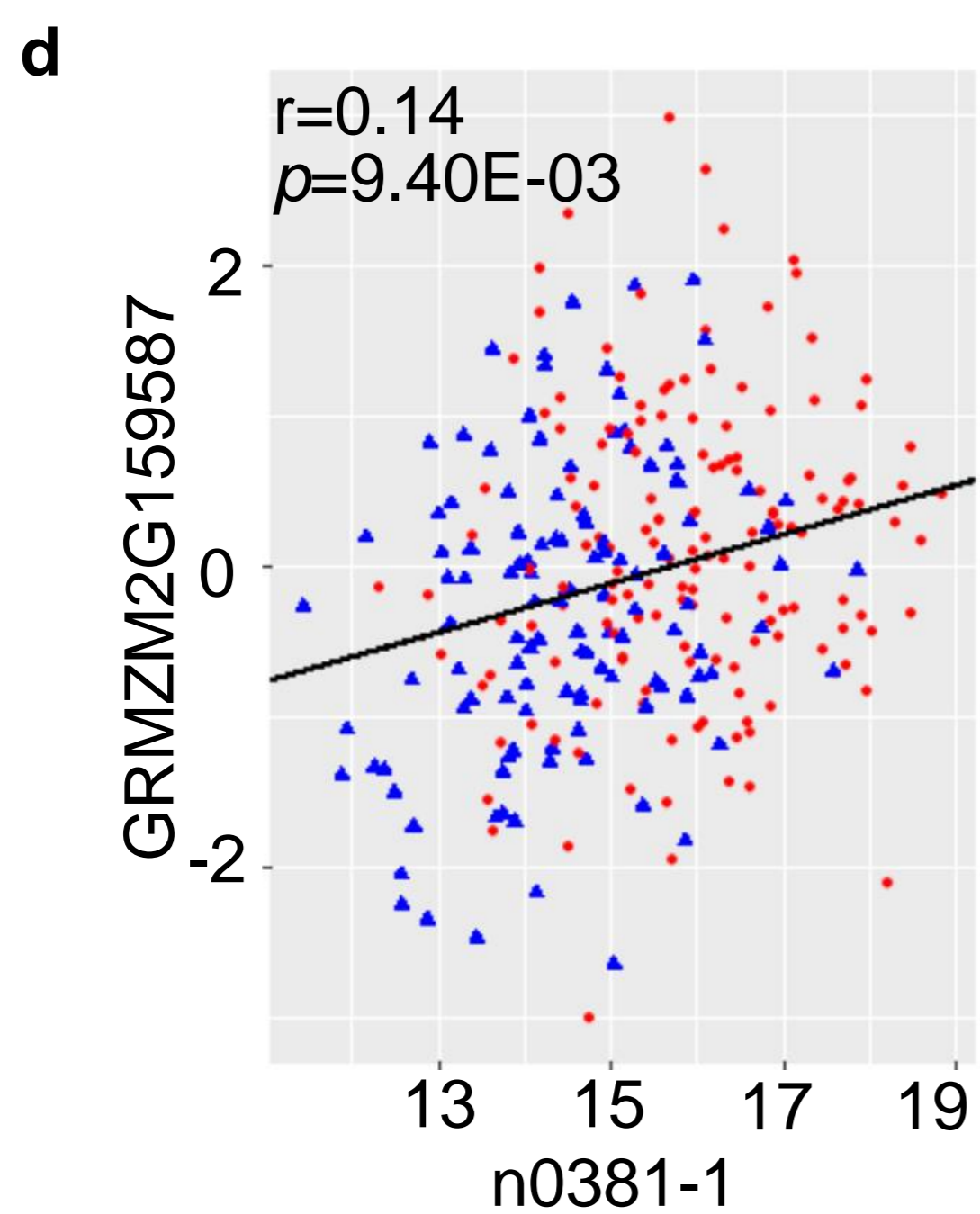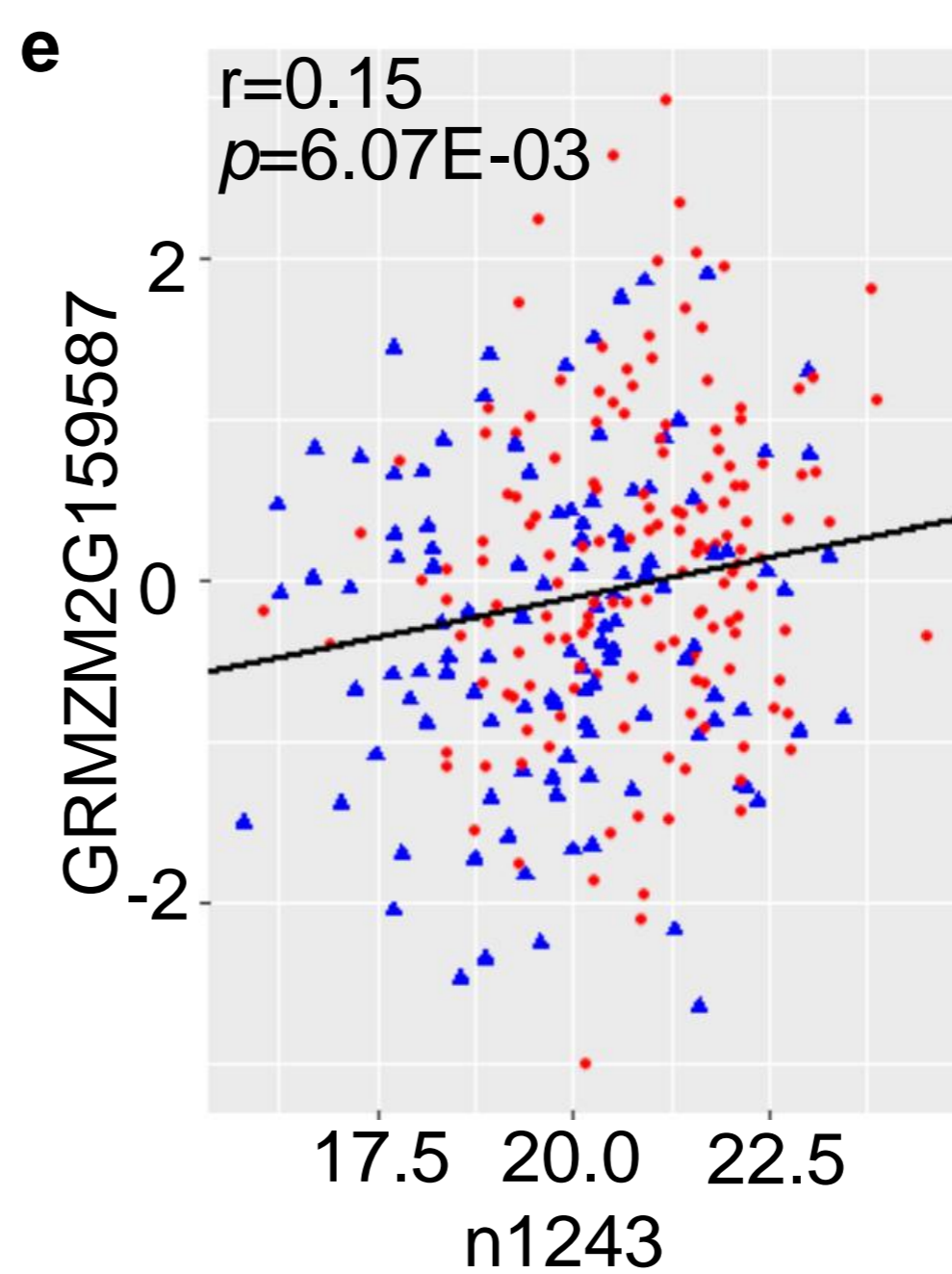

Supplement: Supplementary Figure 7 — GWAS for n0381-1 and n1243 with significant SNP-trait association in this study. (A) Box plot for the n0381-1 level. (B) Box plot for the n1243 level. (C) Box plot for the expression of ZmGR (GRMZM2G159587). (D) Plot of the correlation between the n0381-1 level and the normalized expression level of ZmGR (sky blue). (E) Plot of the correlation between the n1243 level and the normalized expression level of ZmGR (sky blue). The r value is based on a Pearson correlation coefficient. The P value was calculated using Student’s t test. [file Image_7.pdf]
